# Recommendations for the management of severe malaria and severe dengue in resource-limited settings

**DOI:** 10.1007/s00134-016-4602-2

**Published:** 2016-11-05

**Authors:** Arjen M. Dondorp, Mai Nguyen Thi Hoang, Mervyn Mer, for the Sepsis in Resource-Limited Settings-Expert Consensus Recommendations Group of the European Society of Intensive Care Medicine (ESICM) and the Mahidol-Oxford Research Unit (MORU) in Bangkok, Thailand

**Affiliations:** 1 Mahidol-Oxford Research Unit (MORU), Faculty of Tropical Medicine, Mahidol University, Bangkok, 10400 Thailand; 2 Oxford Centre for Tropical Medicine and Global Health, Nuffield Department of Clinical Medicine, University of Oxford, Oxford, UK; 3 Department of Intensive Care, Academic Medical Center, University of Amsterdam, Amsterdam, The Netherlands; 4 Oxford University Clinical Research Unit, Hospital for Tropical Diseases, Ho Chi Minh City, Vietnam; 5 Department of Critical Care, Johannesburg Hospital and University of the Witwatersrand, Johannesburg, South Africa

## Introduction

Sepsis in resource-limited settings will often have different aetiologies to those in western settings, including severe malaria, severe dengue, viral haemorrhagic fevers, mellioidosis, typhus, and leptospirosis. The Surviving Sepsis Campaign (SSC) guidelines [1] are mainly based on evidence from studies on bacterial sepsis. These guidelines are widely applicable, but there are also exceptions. We focus here on disease-specific recommendations for the management of severe falciparum malaria and severe dengue. An international team with extensive practical experience in resource-limited intensive care units (ICUs) identified key questions concerning the SSC’s management recommendations on these diseases. Pertinent evidence from resource-limited settings was evaluated using the grading of recommendations assessment, development and evaluation (GRADE) tools.

### Recommendations for the management of severe malaria and severe dengue in resource-limited settings

#### Severe falciparum malaria

Severe falciparum malaria is a multi-organ disease caused by *Plasmodium falciparum* transmitted by *Anopheles* mosquitoes. The highest transmission and disease burden is in sub-Saharan Africa, where severe malaria is largely a paediatric disease, as older children and adults become partly immune. In Asia and South America, all age groups may be affected. Independent of age, the presenting symptoms with the strongest prognostic significance are coma (cerebral malaria), metabolic (lactic) acidosis and renal dysfunction. Hypotension occurs infrequently (~12% of cases). One of the main pathophysiologic differences of severe falciparum malaria compared to bacterial sepsis is microcirculatory impairment caused by sequestration of parasite-infected erythrocytes, red cell rigidity and red cell clumping. Management requires rapid parasitological diagnosis by microscopy or rapid diagnostic testing (RDT) and prompt initiation of parenteral artesunate [2]. The SSC recommends that, in patients with sepsis-induced tissue hypoperfusion and suspicion of hypovolemia, with either hypotension or hyperlactatemia, an initial fluid challenge of at least 30 ml/kg of crystalloids be administered, of which a portion may be albumin equivalent [1]. Both paediatric and adult patients with severe malaria and tissue hypoperfusion are volume depleted intravascularly. A large trial on fluid bolus therapy in 3138 African children with severe infections and compensated shock, 57% of whom had falciparum malaria, showed an overall 40% increase in mortality with fluid bolus therapy (20 or 40 ml/kg with either saline or 5% albumin). In the 1793 children with severe *P. falciparum* malaria, mortality in the bolus groups was 51% higher [RR 1.51 (1.17–1.95)] [3]. In Asian studies of adult severe malaria, rapid fluid resuscitation did not improve metabolic acidosis [4, 5] and transpulmonary thermodilution-guided rapid fluid resuscitation resulted in pulmonary oedema in 8/28 (29%) patients [5]. One observational study from Myanmar showed no deterioration in renal function or plasma lactate with maintenance fluid therapy between 1.3 and 2.2 ml/kg/h [6]. We recommend against the use of fluid bolus therapy in normotensive patients with severe falciparum malaria (1A). In normotensive patients, we suggest initial (24 h) crystalloid fluid therapy of 2–4 ml/kg/h, which may subsequently be reduced to 1 ml/kg/h in patients receiving additional fluids, e.g. through enteral tube feeding (2D). We suggest fluid bolus therapy (30 ml/kg) with an isotonic crystalloid in patients with hypotensive shock and, if available, early initiation of vasopressor support (ungraded) (Table 1).

The SSC suggests administering enteral feeding within the first 48 h after diagnosing severe sepsis. In resource-limited settings, endotracheal intubation of comatosed patients is often not practised. In a randomised trial in non-intubated predominantly adult Bangladeshi patients with cerebral malaria, early enteral feeding (<36 h), was associated with aspiration pneumonia in 9/27 (33%) as compared to 0/29 when feeding was commenced after 60 h [7]. No difference in hypoglycaemia incidence was observed. We suggest initiating enteral feeding in non-intubated adult patients with cerebral malaria after 60 h (2B). There are insufficient data on paediatric patients with cerebral malaria from African settings. For patients with sepsis-induced respiratory failure, low tidal volume (6 mL/kg) mechanical ventilation is recommended conform the SSC. In patients with cerebral malaria, we suggest against the use of permissive hypercapnia to achieve this goal, since this may exacerbate brain swelling (ungraded).

#### Severe dengue

Severe dengue is caused by dengue virus transmitted by *Aedes* mosquitoes. Approximately 1–5% of patients will develop severe manifestations. The defining feature is a vasculopathy with increased capillary permeability, causing plasma leakage, reduced intravascular volume and, if severe, life-threatening hypovolemic shock [8]. This ‘critical phase’ typically starts during the period of defervescense, and lasts for approximately 48 h. Bleeding complications and organ involvement of the brain, liver, kidney and heart may be additional features, and occur more frequently in adult cases [9]. Diagnosis is commonly with combined dengue antigen (NS1) and antibody RDT [8]. No antiviral treatment is currently available (Table 1).

Unlike in bacterial sepsis, capillary leak in patients with severe dengue results in haemoconcentration. Haemorrhage, in particular from the gut, can contribute to hypovolemic shock [9]. Myocarditis is rare, but some depression of myocardial contractility is common. The World Health Organisation (WHO) guidelines on fluid resuscitation recommend restoration of the circulation guided by pulse pressure, capillary refill time, haematocrit and urine output [8]. Cautious but prompt fluid administration is essential and should be restricted as soon as the critical phase is over to avoid pulmonary oedema [10]. We recommend fluid administration according to the WHO guidelines (1C). We recommend that rapid (<30 min) administration of large (>15 ml/kg) fluid boluses are avoided, unless the patient is hypotensive (1D). In patients with compensated shock, colloids are not superior to normal saline or Ringer’s lactate for shock reversal, or for the prevention of recurrent shock, and we recommend that colloids are not used (1A) [11–13]. There is insufficient evidence to recommend fluid choice in dengue hypotensive shock. The use of corticosteroids is not recommended (1B) [14]. Although thrombocytopenia is an inherent feature of severe dengue, the cause of bleeding is multifactorial, including a prominent vasculopathy. Bleeding is not prevented by platelet transfusion [15]; we do not recommend platelet transfusion for thrombocytopenia in the absence of active bleeding complications or other risk factors (1C). In cases of bleeding complications, we suggest transfusion of fresh-frozen plasma (or cryoprecipitate) and platelet concentrate (ungraded).

## Conclusions

The management of severe malaria and severe dengue differs in some important aspects from the treatment of bacterial sepsis, in particular, regarding fluid management.

**Table 1:** Recommendations and suggestions for the management of patients with severe malaria and severe dengue in resource-limited settings (with grading)

Fluid management of severe malaria	We recommend not to use fluid bolus therapy in normotensive patients with severe falciparum malaria (1A). We suggest that patients receive maintenance isotonic crystalloid fluid therapy (2–4 ml/kg/h), which may subsequently be reduced to 1 ml/kg/h in patients receiving additional fluids, e.g. through enteral tube feeding (2D). We suggest that, in patients with hypotensive shock, fluid bolus therapy (30 ml/kg) with isotonic crystalloids be commenced (ungraded) and, if available, early initiation of vasopressor medication (ungraded)
Timing of enteral feeding in cerebral malaria	We suggest initiating enteral feeing in non-intubated adult patients with cerebral malaria after 60 h, in order to limit the possibility of aspiration pneumonia (2B). There are insufficient data to make this recommendation for children with cerebral malaria
Permissive hypercapnia in ventilated cerebral malaria	We suggest not to use a strategy of permissive hypercapnia to achieve ventilation with low tidal volumes in patients with cerebral malaria, because of the high incidence of brain swelling in these patients (ungraded)
Fluid management in severe dengue	We recommend that fluid resuscitation in severe dengue is executed promptly and guided by pulse pressure, capillary refill time, haematocrit and urine output according to WHO guidelines, and that fluid therapy should be restricted as soon as the critical phase of the disease is over to avoid pulmonary oedema (1C). We recommend that rapid administration of large fluid boluses should be avoided, unless the patient is hypotensive (1D). We recommend that, in dengue patients with compensated shock, colloid fluids are not used (1A)
Use of corticosteroids in severe dengue	We recommend not to use corticosteroids in the treatment of severe dengue (1B)
Use of prophylactic platelet transfusion in severe dengue	We recommend not to use prophylactic platelet transfusion for thrombocytopenia in the absence of active bleeding complications, or other risk factors (uncontrolled arterial hypertension, recent stroke, head trauma or surgery, continuation of an anticoagulant treatment, or existing haemorrhagic diathesis) (1B)

## Electronic supplementary material

Below is the link to the electronic supplementary material.

Supplementary material 1 (DOCX 88 kb)
