# Supplementary material for: Recommendations for the management of severe malaria and severe dengue in resource-limited settings
Source: Intensive Care Med. 2016 Nov 5;43(11):1683–5. doi: 10.1007/s00134-016-4602-2 (PMC5633627; doi:10.1007/s00134-016-4602-2)
Supplement: Supplementary file 1 — Supplementary material 1 (DOCX 88 kb) [file 134_2016_4602_MOESM1_ESM.docx]

Online Supplement to

**Recommendations for management of severe malaria and severe dengue in resource-limited settings**

Arjen M. Dondorp^1,2,3^, Mai Nguyen Thi Hoang^4^, Meryn Mer^5^ for the sepsis in resource-limited settings–expert consensus recommendations group of the European Society of Intensive Care Medicine (ESICM) and the Mahidol-Oxford Research Unit (MORU) in Bangkok, Thailand

**Faculty of Tropical Medicine, Mahidol University, Bangkok, Thailand**

^1^Mahidol–Oxford Research Unit (MORU)

**Nuffield Department of Clinical Medicine, University of Oxford, Oxford, UK**

^2^Oxford Centre for Tropical Medicine and Global Health

**Academic Medical Center, University of Amsterdam, Amsterdam, The Netherlands**

^3^Department of Intensive Care

**Hospital for Tropical Diseases, Ho Chi Minh City, Vietnam**

^4^Oxford University Clinical Research Unit

**Johannesburg Hospital and University of the Witwatersrand, Johannesburg, South Africa**

^5^Department of Critical Care

| **Correspondence:**  Prof. Arjen M Dondorp, MD  Mahidol Oxford Tropical Medicine Research Unit  Faculty of Tropical Medicine, Mahdiol University  420/6 Rajvithi Road, Bangkok 10400, Thailand  E-mail: Arjen@tropmedres.ac | **Email of other authors:**  Mai Nguyen Thi Hoang: Mainth@oucru.org  Mervyn Mer: Mervyn.Mer@wits.ac.za |
| --- | --- |

**Group members of the ‘Sepsis in Resource–limited Settings’–guidelines group**

(*Heads*) Arjen Dondorp (Faculty of Tropical Medicine, Mahidol University, Bangkok,Thailand & Academic Medical Center, University of Amsterdam, Amsterdam, The Netherlands &), Martin Dünser (Department of Critical Care, University College of London Hospital, London, United Kingdom), and Marcus Schultz (Academic Medical Center, University of Amsterdam, Amsterdam, The Netherlands & Faculty of Tropical Medicine, Mahidol University, Bangkok, Thailand). *Members of the malaria-dengue core writing group in addition to listed authors*: Sanjib Mohanty (Ispat General Hospital, Rourkela, Sundargarh, Odisha, India), Marcus Schultz (Academic Medical Center,University of Amsterdam, Amsterdam, The Netherlands & Faculty of Tropical Medicine,Mahidol University, Bangkok, Thailand), Louise Thwaites (Centre for Tropical Medicine and Global Health, Nuffield Department of Medicine, University of Oxford, UK), Martin Dünser (Department of Critical Care, University College of London Hospital, London, United Kingdom), Jane Nakibuuka (Mulago National Referral and University Teaching Hospital, Kampala, Uganda). (*Other group members, in alphabetic order*) Neill K.J. Adhikari (Sunnybrook Health Sciences Centre & University of Toronto, Toronto, ON, Canada), Derek Angus (University of Pittsburgh, Pittsburgh, PA), Luciano Azevedo (Hospital Sirio–Libanes, Saõ Paulo, Brazil), Kwizera Arthur (Mulago National Referral Hospital, Kampala, Uganda),Timothy Baker (Karolinska Institute, Stockholm, Sweden), Ted Barnett (The Ochsner Medical Center, New Orleans, Louisiana), Chris Farmer (Mayo Clinic, Rochester, MI),Rashan Haniffa (Faculty of Tropical Medicine, Mahidol University, Bangkok, Thailand),Randeep Jawa (Stony Brook University Medical Center, Stony Brook, NY, USA),Niranjan Kissoon (British Columbia Children’s Hospital and University of British Columbia, Vancouver, Canada), Rakesh Lodha (All India Institute of Medical Science,Delhi, India), Ganbold Lundeg (Mongolian National University of Medical Sciences,Ulaanbaatar, Mongolia), Ignacio Martin Loeches (St. James's University Hospital,Dublin, Ireland), David Misango (Aga Khan University Hospital, Nairobi, Kenya), Mervyn Mer (Johannesburg Hospital and University of the Witwatersrand, Johannesburg, South Africa), ,Srinivas Murthy (BC Children’s Hospital, University of British Columbia, Vancouver,Canada), Ndidiamaka Musa (Seattle Children’s Hospital and University of Washington,WA), Jane Nakibuuka (Mulago National Referral and University Teaching Hospital, Kampala, Uganda), Mai Nguyen Thi Hoang (Oxford University Clinical Research Unit,Ho Chi Minh City, District 5, Vietnam), Binh Nguyen Thien (Trung Vuong Hospital, Ho Chi Minh City, Viet Nam), Rajyabardhan Pattnaik (Ispat General Hospital, Rourkela,Sundargarh, Odisha, India), Luigi Pisani (University of Bari Aldo Moro, Bari, Italy), Jason Phua (Yong Loo Lin School of Medicine, National University of Singapore, Singapore,Singapore), Jacobus Preller (Addenbrooke’s Hospital, Cambridge University Hospitals NHS Foundation Trust, Cambridge, UK), Pedro Povoa (Nova Medical School, CEDOC,New University of Lisbon, Lisbon, Portugal & Hospital de São Francisco Xavier, Centro Hospitalar de Lisboa Ocidental, Lisbon, Portugal), Suchitra Ranjit (Appolo’s Hospitals, Chennai, India), Jonarthan Thevanayagam (Mzuzu Central Hospital, Mzuzu, Malawi).

**Guideline development**

*Selection of group members*

The selection of the group members was based on interest in specific aspects of sepsis and experience in intensive care units (ICUs) in resource–limited settings. Potential team members were contacted via email and in person at the ‘27th Annual Congress of the European Society of Intensive Care Medicine’ in Barcelona, Spain from 27 September – 1 October 2014, and the ‘35th International Symposium on Intensive Care and Emergency Medicine’ in Brussels, Belgium from 17 March – 20 March 2015, and nine subgroups were created, assigned to nine areas in sepsis management, i.e.,‘diagnosis of sepsis’, ‘levels of ICU’, ‘organizational aspects’, ‘management of infection’, ‘hemodynamic support’, ‘ventlatory support’, ‘sedation, renal failure, prophylaxes, glucose control and feeding’, ‘tropical aspects’ and ‘pediatric aspects’. Additional team members were appointed by the group heads to address content needs for the development process. Several group members had experience in ‘Grading of Recommendations Assessment, Development and Evaluation’ (GRADE) process and use of the GRADE pro Guideline Development Tool[[1](#_ENREF_1)].

*Selection of subgroup heads and subgroup members*

Arjen Dondorp Mai Nguyen Thi Hoang and Martin Dünser were appointed as the group heads; other members were assigned to this subgroup based on their specific expertise and interest in management of infection.

*Question identification*

Key questions regarding “Recommendations for the management of severe malaria and severe dengue in resource-limited settings”, based on the SSC recommendations were identified by the subgroup. Priority was given to areas where particular differences between resource-limited and resource-rich settings were likely to arise, or where current recommendations may impose particular burdens on resources.

*Literature review*

Subgroup members primarily searched for additional articles from resource-limited settings relevant to the questions identified, in a minimum of one general database (i.e.,MEDLINE, EMBASE) and the Cochrane Libraries. Relevant articles that were detected through citations within articles were also included. Searches were confined to studies examining adult populations unless otherwise stated.

*Grading of Recommendations*

The subgroup members also followed the principles of the GRADE process as

described for the development of the Surviving Sepsis Campaign’–guidelines[[1](#_ENREF_1), [2](#_ENREF_2)]. In short, GRADE classifies quality of evidence as high (grade A), moderate (grade B), low (grade C), or very low (grade D) and recommendations as strong (grade 1) or weak (grade 2). A strong recommendation is worded as ‘*we recommend*’ and a weak recommendation as ‘*we suggest*’. A number of recommendations could remain ‘ungraded’, when, in the opinion of the subgroup members, such recommendations were not conducive for the GRADE process.

*Definitions*

During preparation of this manuscript, The European Society of Intensive Care Medicine’s and the Society of Critical Care Medicine’s Consensus Definitions for Sepsis and Septic Shock (Sepsis-3) were published[[3](#_ENREF_3)]. For the purposes of this article, where published studies have used former definitions of severe sepsis and septic shock to characterize patients, these have been left as originally published. For further clarification, we refer readers to the Surviving Sepsis Campaign and the article in this series examining sepsis recognition[[4](#_ENREF_4), [5](#_ENREF_5)]. We have defined resource-limited settings as those within countries defined as low or middle income countries according to World Bank[[6](#_ENREF_6)] or described as ‘resource-limited’ or ‘developing countries’ by authors of studies themselves.

*Conflicts of interest*

No members of the nine subgroup represented industry, and there was no industry input into guidelines development. No member of the nine subgroup received honoraria for any role in the guideline development process. Each member provided a standard COI–form, to be uploaded through the GRADEpro Guideline Development Tool website.

**Questions**

Six questions regarding the management of severe malaria and severe dengue were formulated:

Question 1. How much and which intravenous fluids should be used in patients with severe malaria and signs of shock?

Question 2. Should patients with cerebral malaria receive early enteral nasogastric tube feeding?

Question 3. Is permissive hypercapnia a suitable strategy to achieve low tidal volume mechanical ventilation in patients with cerebral malaria?

Question 4. How much and which intravenous fluids should be used in patients with severe dengue/ dengue shock syndrome?

Question 5. Should corticosteroids be used in patients with severe dengue?

Question 6. Should preventive platelet transfusion be used in patients with severe dengue?

**Detailed results**

*Question 1.* How much and which intravenous fluids should be used in patients with severe malaria and signs of shock?

*Rationale.* Severe malaria is an old disease, and historically, the guidance for fluid management has been to ‘keep them dry’. This approach was subsequently challenged when it was recognized that severe malaria is a severe sepsis syndrome with signs of tissue hypoperfusion, and thus might benefit from fluid bolus therapy. The SSC recommends in patients with sepsis-induced tissue hypoperfusion and suspicion of hypovolemia an initial fluid challenge of minimal 30 mL/kg of crystalloids, to be completed within 3 hours, of which a portion may be albumin equivalent; this applies to patients with hypotension or a plasma lactate ≥ 4 mmol/L[[2](#_ENREF_2)]. It was shown by various techniques that both children and adults with severe falciparum malaria are intravascular dehydrated[[7-9](#_ENREF_7)] although this was debated by some[[10](#_ENREF_10)].

*Evidence.* Small trials in African children with severe malaria suggested a benefit from fluid bolus therapy, in particular with albumen[[11-14](#_ENREF_11)], reviewed by Akech et al in 2010[[15](#_ENREF_15)]. However, a subsequent large trial on fluid bolus therapy in 3138 African children with severe infections and compensated shock, of which 57% had falciparum malaria, showed overall a 40% *in*crease in mortality with fluid bolus therapy (20 ml/kg or 40 ml/kg with either saline or albumin). In the 1793 children with severe *P. falciparum* malaria, mortality in the bolus groups was 51% higher [(RR 1.51(1.17-1.95)] than without fluid bolus therapy[[16](#_ENREF_16)]. In the same study, febrile patients with hypotensive (“decompensated’) shock were randomised between 20 to 40 ml/kg fluid bolus therapy with either saline or albumin; 69% of the children (9 of 13) in the albumin bolus group and 56% (9 of 16) in the saline-bolus group died (P = 0.45). In Asian studies in adult severe malaria, rapid fluid resuscitation did not improve metabolic acidosis[[17](#_ENREF_17), [18](#_ENREF_18)] and transpulmonary thermodilution-guided rapid fluid resuscitation resulted in pulmonary oedema in 8/28 (29%) patients[[18](#_ENREF_18)] . One observational study showed no deterioration in renal function or plasma lactate with maintenance fluid therapy between 1.3-2.2 ml/kg/hour[[19](#_ENREF_19)]. A recent systematic review concluded that fluid bolus therapy with either crystalloid or albumen is not beneficial in severe falciparum malaria[[20](#_ENREF_20)]. We recommend not to use fluid bolus therapy in normotensive patients with severe falciparum malaria (1A). We suggest not to use colloid therapy, including albumin 5% (2C). In normotensive patients, we suggest initial crystalloid fluid therapy of 2 to 4 ml/kg/hour (2D). In patients receiving enteral fluids, e.g. through enteral tube feeding, we suggest that this can be reduced to 1 ml/kg/hour (2D). This is slightly more conservative that the recommendation in the management guidelines for severe malaria issued by the World Health Organisation, recommending 3-5 ml/kg/hour[[21](#_ENREF_21)]. There are no data on the benefit of balanced fluids over normal saline. We suggest fluid bolus therapy (30 ml/kg) with an isotonic crystalline in patients with hypotensive shock, and if available early start of vasopressive medication (ungraded).

*Question 2*. Should patients with cerebral malaria receive early enteral nasogastric tube feeding?

*Rationale*. The SSC suggest administering oral or enteral (if necessary) feeds, as tolerated, rather than either complete fasting or provision of only intravenous glucose within the first 48 h after a diagnosis of severe sepsis/septic shock (grade 2C)[[2](#_ENREF_2)]. Early enteral feeding is thought to preserve gut integrity and function, maintain bile secretion and secretory IgA, maintain gut associated lymphoid tissue (GALT) resulting in reduced translocation, improve splanchnic blood flow, and to act prophylactically against stress ulceration. In patients with severe malaria, malnutrition is common, as is concomitant invasive bacterial infection[[22](#_ENREF_22)]. Therefore, the recommendation for early start of enteral feeding seems valid for patients with severe malaria, including intubated patients with cerebral malaria. However, in resource-limited settings endotracheal intubation of comatose patient is often not practiced, and there might be an increased risk of aspiration pneumonia.

*Evidence.* We could identify one randomised trial on the timing of enteral feeding in patients with cerebral malaria[[23](#_ENREF_23)]. This trial in (mainly) adult Bangladeshi patients with cerebral malaria who were not on mechanical ventilation, and thus had an unprotected airway, showed that early (<60 hours) enteral feeding was associated with aspiration pneumonia in 9/27 (33%) versus 0/29 with late start after 60 hours (p=0.001). This despite proper positioning of patients, and pre-feed inspection of gastric retention. No difference in the incidence of hypoglycemia was observed. We suggest starting enteral feeding in non-intubated adult patients with cerebral malaria after 60 hours (2B). There are insufficient data on paediatric patients with cerebral malaria from African settings.

*Question 3.* Is permissive hypercapnia a suitable strategy to achieve low tidal volume mechanical ventilation in patients with cerebral malaria?

*Rationale.* Acute Respiratory Distress Syndrome (ARDS), or pulmonary malaria, is a feared complication of severe falciparum malaria, and can also complicate the course of vivax malaria[[24](#_ENREF_24)]. The incidence of ARDS in adult patients with severe malaria is estimated 5%-25% and up to 29% in pregnant women; ARDS is thought to be rare in paediatric severe malaria[[25](#_ENREF_25)]. To protect the lung from the damaging effects of mechanical ventilation, the SSC recommends targeting a tidal volume of 6 mL/kg predicted body weight in patients with sepsis-induced acute respiratory distress syndrome (ARDS), and that plateau pressures be measured in patients with ARDS and that the initial upper limit goal for plateau pressures in a passively inflated lung be <30 cmH_2_O[[2](#_ENREF_2)]. There are no randomised trials to evaluate this recommendation specifically for ARDS in the context of severe malaria. However, given the large benefit of this ventilation strategy in patients with other causes of ARDS, this recommendation should also be valid in severe malaria. The SSC guidelines also suggest that to facilitate use of a lung protective ventilatory strategy, permissive hypercapnia can be used. This might not be appropriate in patients with cerebral malaria because of potential brain swelling. In addition, availability of blood gas or End-tidal pCO2 monitoring is limited in resource-poor settings, compromising its safe implementation.

*Evidence.* There are no randomised trials on the use of permissive hypercapnia in mechanically ventilated patients with severe falciparum malaria. However, in cerebral malaria brain swelling is common, caused by an increase in intracerebral blood volume including the sequestered parasitized red blood cell-mass, vasogenic oedema, and cytotoxic oedema, and is more prominent in paediatric cases[[26-29](#_ENREF_26)]. Because hypercapnia will further increase intracranial pressure we suggest against the use of permissive hypercapnia to achieve the goal of low tidal volume ventilation in patients with cerebral malaria, as cerebral malaria is associated with brain swelling and variably increased intracranial pressure (ungraded).

*Question 4.* How much and which intravenous fluids should be used in patients with severe dengue/ dengue shock syndrome?

*Rationale.* Severe dengue is a sepsis syndrome. Yet, important aspects of the pathophysiology of the circulatory changes are distinct from bacterial sepsis. Dengue shock syndrome is characterised by a vasculopathy during the critical phase of the disease, with a plasma leak and haemoconcentration, causing important intravascular volume depletion[[30](#_ENREF_30)]. This initially leads to a compensated shock with signs of tissue hypoperfusion and a decreased pulse pressure with preserved systolic blood pressure. This can be followed by life-threatening hypotensive shock. Haemorrhage, in particular from the gastrointestinal tract, and more rarely myocarditis, can contribute to circulatory shock. The onset is usually more gradual than with bacterial sepsis. Management of patients with severe dengue relies largely on careful monitoring, including early recognition of vascular leakage and proper fluid replacement, combined with prompt but carefully guided volume resuscitation for patients who develop dengue shock syndrome. The SSC guidelines advocate fluid bolus therapy for patients with sepsis-induced tissue hypoperfusion and suspicion of hypovolemia[[2](#_ENREF_2)], which might not be appropriate for patients with severe dengue and compensated shock. In addition, because of the prominent plasma leak, the use of colloids might be beneficial in severe dengue, as opposed to its use in patients with bacterial sepsis. The WHO guidelines for the management of patients with severe dengue distinguish patients with compensated shock from those with decompensated (hypotensive) shock[[31](#_ENREF_31), [32](#_ENREF_32)]. In compensated shock, recommended initial fluid therapy is with isotonic crystalloid solutions at 5–10 ml/kg over one hour, which can be tapered every few hours if the patient improves guided by the pulse pressure, capillary refill time, haematocrit, and urine output. Prudential fluid therapy is important throughout the disease, but in particular fluid administration should be restricted as soon as the critical phase of the disease is over to avoid pulmonary oedema. In the same guidelines, it is recommended in patients with hypotensive shock, to resuscitate with crystalloid or colloid solution at 20 ml/kg as a bolus given over 15 minutes.

*Evidence.*

No randomised clinical trials to support the fluid resuscitation recommendations could be identified. Fluid bolus therapy, and liberal fluid management more in general, was a risk factor for respiratory distress in a large prospective observational study in Latin American and Asian patients with dengue[[33](#_ENREF_33)]. A large prospective observational study in 1719 Vietnamese children with laboratory-confirmed dengue shock syndrome practice an initial fluid regimen of Ringer’s lactate solution at 25 mL/kg over 2 hours, with colloid solutions reserved for children presenting with decompensated shock[[34](#_ENREF_34)]. The observed case fatality rate with this approach was 8/1719 children (0.5%). We recommend to follow the current WHO guidelines on fluid management in severe dengue/ dengue shock syndrome (1C). We recommend that rapid (<30 min) administration of large (>15 ml/kg) fluid boluses should be avoided, unless the patient is hypotensive (1D).

There are several randomised trials comparing crystalloid with colloid fluid management for the treatment of patients with severe dengue and compensated shock. In a Vietnamese trial 383 children with moderately severe dengue shock syndrome were randomised to fluid therapy with either Ringer’s lactate, 6% dextrose or 6% hydroxyethyl starch in a 1:1:1 ratio[[35](#_ENREF_35)]. Need for rescue resuscitation with a colloid or the proportion of children with shock recurrence (which carries a worse prognosis) was similar between treatment arms. An additional 2 other randomised trials did not show better outcome parameters with (more expensive) colloids over crystalloid fluids[[36](#_ENREF_36), [37](#_ENREF_37)]. A quasi randomised study from the Philippines with alternate allocation of starch versus crystalloid fluids also did not show an additional benefit of colloid therapy[[38](#_ENREF_38)]. We recommend that in dengue patients with compensated shock colloids are not used for initial resuscitation (1A). There is insufficient evidence to recommend fluid choice in severe dengue with hypotensive shock, but there is discussion among experts whether there is in fact still a role for colloids in severe dengue patients with hypotension, given the prominent role of capillary leak it its pathogenesis. Since current evidence strongly suggests that all hydroxyethyl starches (HES) increase the risk of acute kidney injury and renal replacement therapy[[39](#_ENREF_39)], we suggest not to use HES for fluid resuscitation in severe dengue patient with severe dengue (ungraded).

*Question 5.* Should corticosteroids be used in patients with severe dengue?

*Rationale.* Both humoral and cellular immune responses are thought to be implicated in the pathogenesis of vasculopathy, which is central in the pathogenesis of dengue shock syndrome[[40](#_ENREF_40)]. The risk for developing severe disease is increased in secondary heterotypic infections, in which antibody dependent enhancement (ADE) of infection and cross-reactive memory T cells are thought to play a role. These insights have led to the use of immunomodulatory therapy with corticosteroids in severe dengue infection.

*Evidence.* A Cochrane review on patients with dengue shock syndrome identified 4 randomised or quasi-randomised trials comparing corticosteroids with no corticosteroids or placebo involving 284 participants with dengue shock syndrome[[41](#_ENREF_41)]. Corticosteroids did not reduce the number of deaths (RR 0.68, 95% CI 0.42 to 1.11; 284 participants, 4 trials), the need for blood transfusion (RR 1.08, 0.52 to 2.24; 89 participants, 2 trials), or the number of serious complications (convulsions and pulmonary haemorrhage, 1 trial). The evidence was rated low quality as most studies were underpowered or lacked stringent randomization or allocation concealment. Corticosteroids were administered after the onset of shock. A more recent Vietnamese randomised trial in 225 children with dengue fever evaluated early oral prednisolone therapy (2 mg/kg versus 0.5 mg/kg versus placebo for 3 days)[[42](#_ENREF_42)]. Use of oral prednisolone was not associated with prolongation of viraemia and was considered safe. However, no reduction in the development of dengue shock syndrome or other complication were observed with early prednisolone therapy, although the trial was not sufficiently powered to assess efficacy. An additional analysis of the same trial focusing on immunological endpoints did not show an important attenuation of the host immune response with prednisolone treatment[[43](#_ENREF_43)]. A additional Cochrane review of trials on the early use of corticosteroids in patients with dengue fever identified 4 studies (including the study discussed above), enrolling a total 664 children and adults, showing no benefit of corticosteroids regarding mortality or dengue complications, although the evidence was considered low to very low quality[[44](#_ENREF_44)]. With the current level of evidence the use of corticosteroids is not recommended in the treatment of severe dengue (1B).

*Question 6.* Should preventive platelet transfusion be used in patients with severe dengue?

*Rationale.* Bleeding is a feared complication of severe dengue infection. Thrombocytopenia with a thrombopathy is invariably present in patients with severe dengue infection. However, vasculopathy is a central and important additional contributor to the bleeding risk[[30](#_ENREF_30)]. Prophylactic transfusion of platelets is common practice in dengue endemic countries[[45](#_ENREF_45)]. Platelet transfusion is not without risks, since it can cause allergic reactions and transmission of blood–borne pathogens.

*Evidence.* An open-label randomised study in 87 patients with dengue and a platelet count below 30,000/μl did not show decreased incidence of severe bleeding with prophylactic platelet transfusion[[46](#_ENREF_46)]. A non-randomised Singaporean study in 256 dengue patients with thrombocytopenia <20,000/μL, of whom 188 were given prophylactic platelet transfusion, also did not show decreased bleeding episodes in the treatment group[[47](#_ENREF_47)]. An observational study from Martinique during a dengue outbreak evaluated a conservative strategy to prophylactic platelet transfusion (only if platelets counts <5,000/µL or in case of additional risk factors <20,000/μL). A poor correlation between thrombocytopenia and the occurrence of severe bleeding during admission was observed, and the followed transfusion strategy was considered safe[[48](#_ENREF_48)]. The WHO guidelines do not recommend prophylactic platelet transfusion in severe dengue. The results of the Adult Dengue Platelet Study (ADEPT, ClinicalTrials.gov: NCT01030211), a prospective randomized open-label trial to examine the safety and efficacy of prophylactic platelet transfusion in Singaporean adults with severe dengue-related thrombocytopenia (platelet count below 20,000/μl) but no bleeding, are pending. In resource-poor settings, the availability of safe pathogens-screened blood products can be limited, and platelet transfusion can have important cost implications, supporting restrictive use of platelet transfusion. We do not recommend platelet transfusion for thrombocytopenia in the absence of active bleeding complications or other risk factors such as use of anticoagulants (1C). In case of bleeding complications, we suggest transfusion of fresh-frozen plasma (or cryoprecipitate) and platelet concentrate (ungraded).

**References**

1. (2013) GRADE handbook for grading quality of evidence and strength of recommendations (updated October 2013)

2. Dellinger RP, Levy MM, Rhodes A, Annane D, Gerlach H, Opal SM, Sevransky JE, Sprung CL, Douglas IS, Jaeschke R, Osborn TM, Nunnally ME, Townsend SR, Reinhart K, Kleinpell RM, Angus DC, Deutschman CS, Machado FR, Rubenfeld GD, Webb S, Beale RJ, Vincent JL, Moreno R, (2013) Surviving Sepsis Campaign: international guidelines for management of severe sepsis and septic shock, 2012. Intensive care medicine 39: 165-228

3. Singer M, Deutschman CS, Seymour CW, Shankar-Hari M, Annane D, Bauer M, Bellomo R, Bernard GR, Chiche JD, Coopersmith CM, Hotchkiss RS, Levy MM, Marshall JC, Martin GS, Opal SM, Rubenfeld GD, van der Poll T, Vincent JL, Angus DC, (2016) The Third International Consensus Definitions for Sepsis and Septic Shock (Sepsis-3). Jama 315: 801-810

4. Deutschman CS, Singer M, (2016) Definitions for Sepsis and Septic Shock--Reply. JAMA 316: 458-459

5. Kwizera A, Festic E, Dunser MW, (2016) What's new in sepsis recognition in resource-limited settings? Intensive care medicine

6. Yangzom T, Gueye CS, Namgay R, Galappaththy GN, Thimasarn K, Gosling R, Murugasampillay S, Dev V, (2012) Malaria control in Bhutan: case study of a country embarking on elimination. Malar J 11: 9

7. Yacoub S, Lang HJ, Shebbe M, Timbwa M, Ohuma E, Tulloh R, Maitland K, (2010) Cardiac function and hemodynamics in Kenyan children with severe malaria. Crit Care Med 38: 940-945

8. Maitland K, Levin M, English M, Mithwani S, Peshu N, Marsh K, Newton CR, (2003) Severe P. falciparum malaria in Kenyan children: evidence for hypovolaemia. QJM : monthly journal of the Association of Physicians 96: 427-434

9. Hanson JP, Lam SW, Mohanty S, Alam S, Pattnaik R, Mahanta KC, Hasan MU, Charunwatthana P, Mishra SK, Day NP, White NJ, Dondorp AM, (2013) Fluid resuscitation of adults with severe falciparum malaria: effects on Acid-base status, renal function, and extravascular lung water. Crit Care Med 41: 972-981

10. Planche T, Onanga M, Schwenk A, Dzeing A, Borrmann S, Faucher JF, Wright A, Bluck L, Ward L, Kombila M, Kremsner PG, Krishna S, (2004) Assessment of volume depletion in children with malaria. PLoS Med 1: e18

11. Maitland K, Pamba A, English M, Peshu N, Levin M, Marsh K, Newton CR, (2005) Pre-transfusion management of children with severe malarial anaemia: a randomised controlled trial of intravascular volume expansion. British journal of haematology 128: 393-400

12. Maitland K, Pamba A, English M, Peshu N, Marsh K, Newton C, Levin M, (2005) Randomized trial of volume expansion with albumin or saline in children with severe malaria: preliminary evidence of albumin benefit. Clin Infect Dis 40: 538-545

13. Akech S, Gwer S, Idro R, Fegan G, Eziefula AC, Newton CR, Levin M, Maitland K, (2006) Volume expansion with albumin compared to gelofusine in children with severe malaria: results of a controlled trial. PLoS clinical trials 1: e21

14. Maitland K, Pamba A, Newton CR, Levin M, (2003) Response to volume resuscitation in children with severe malaria. Pediatric critical care medicine : a journal of the Society of Critical Care Medicine and the World Federation of Pediatric Intensive and Critical Care Societies 4: 426-431

15. Akech S, Ledermann H, Maitland K, (2010) Choice of fluids for resuscitation in children with severe infection and shock: systematic review. BMJ 341: c4416

16. Maitland K, Kiguli S, Opoka RO, Engoru C, Olupot-Olupot P, Akech SO, Nyeko R, Mtove G, Reyburn H, Lang T, Brent B, Evans JA, Tibenderana JK, Crawley J, Russell EC, Levin M, Babiker AG, Gibb DM, (2011) Mortality after fluid bolus in African children with severe infection. N Engl J Med 364: 2483-2495

17. Nguyen HP, Hanson J, Bethell D, Nguyen TH, Tran TH, Ly VC, Pham PL, Dinh XS, Dondorp A, White N, Day N, (2011) A retrospective analysis of the haemodynamic and metabolic effects of fluid resuscitation in Vietnamese adults with severe falciparum malaria. PLoS One 6: e25523

18. Hanson J, (2012) Fluid resuscitation of adults with severe falciparum malaria: effects on acid-base status, renal function, and extravascular lung water. Critical Care Medicine in press

19. Aung NM, Kaung M, Kyi TT, Kyaw MP, Min M, Htet ZW, Anstey NM, Kyi MM, Hanson J, (2015) The Safety of a Conservative Fluid Replacement Strategy in Adults Hospitalised with Malaria. PLoS One 10: e0143062

20. Hodgson SH, Angus BJ, (2016) Malaria: fluid therapy in severe disease. BMJ clinical evidence 2016

21. WHO, (2014) Severe Malaria. Trop Med Int Health 19: 7-131

22. Berkley JA, Bejon P, Mwangi T, Gwer S, Maitland K, Williams TN, Mohammed S, Osier F, Kinyanjui S, Fegan G, Lowe BS, English M, Peshu N, Marsh K, Newton CR, (2009) HIV infection, malnutrition, and invasive bacterial infection among children with severe malaria. Clin Infect Dis 49: 336-343

23. Maude RJ, Hoque G, Hasan MU, Sayeed A, Akter S, Samad R, Alam B, Yunus EB, Rahman R, Rahman W, Chowdhury R, Seal T, Charunwatthana P, Chang CC, White NJ, Faiz MA, Day NP, Dondorp AM, Hossain A, (2011) Timing of enteral feeding in cerebral malaria in resource-poor settings: a randomized trial. PloS one 6: e27273

24. White NJ, Pukrittayakamee S, Hien TT, Faiz MA, Mokuolu OA, Dondorp AM, (2014) Malaria. Lancet 383: 723-735

25. Taylor WR, Hanson J, Turner GD, White NJ, Dondorp AM, (2012) Respiratory manifestations of malaria. Chest 142: 492-505

26. Warrell DA, Looareesuwan S, Phillips RE, White NJ, Warrell MJ, Chapel HM, Areekul S, Tharavanij S, (1986) Function of the blood-cerebrospinal fluid barrier in human cerebral malaria: rejection of the permeability hypothesis. Am J Trop Med Hyg 35: 882-889

27. Brown H, Rogerson S, Taylor T, Tembo M, Mwenechanya J, Molyneux M, Turner G, (2001) Blood-brain barrier function in cerebral malaria in Malawian children. Am J Trop Med Hyg 64: 207-213

28. Mohanty S, Mishra SK, Patnaik R, Dutt AK, Pradhan S, Das B, Patnaik J, Mohanty AK, Lee SJ, Dondorp AM, (2011) Brain swelling and mannitol therapy in adult cerebral malaria: a randomized trial. Clin Infect Dis 53: 349-355

29. Seydel KB, Kampondeni SD, Valim C, Potchen MJ, Milner DA, Muwalo FW, Birbeck GL, Bradley WG, Fox LL, Glover SJ, Hammond CA, Heyderman RS, Chilingulo CA, Molyneux ME, Taylor TE, (2015) Brain swelling and death in children with cerebral malaria. N Engl J Med 372: 1126-1137

30. Simmons CP, Farrar JJ, Nguyen v V, Wills B, (2012) Dengue. N Engl J Med 366: 1423-1432

31. WHO (2009) Dengue: Guidelines for treatment, prevention and control. In: Editor (ed)^(eds) Book Dengue: Guidelines for treatment, prevention and control. Geneva: World Health Organization, City, pp.

32. WHO (2012) Handbook for Clinical Management of Dengue. WHO, Geneva

33. Rosenberger KD, Lum L, Alexander N, Junghanss T, Wills B, Jaenisch T, (2016) Vascular leakage in dengue--clinical spectrum and influence of parenteral fluid therapy. Trop Med Int Health 21: 445-453

34. Lam PK, Tam DT, Diet TV, Tam CT, Tien NT, Kieu NT, Simmons C, Farrar J, Nga NT, Qui PT, Dung NM, Wolbers M, Wills B, (2013) Clinical characteristics of Dengue shock syndrome in Vietnamese children: a 10-year prospective study in a single hospital. Clin Infect Dis 57: 1577-1586

35. Wills BA, Nguyen MD, Ha TL, Dong TH, Tran TN, Le TT, Tran VD, Nguyen TH, Nguyen VC, Stepniewska K, White NJ, Farrar JJ, (2005) Comparison of three fluid solutions for resuscitation in dengue shock syndrome. N Engl J Med 353: 877-889

36. Dung NM, Day NP, Tam DT, Loan HT, Chau HT, Minh LN, Diet TV, Bethell DB, Kneen R, Hien TT, White NJ, Farrar JJ, (1999) Fluid replacement in dengue shock syndrome: a randomized, double-blind comparison of four intravenous-fluid regimens. Clin Infect Dis 29: 787-794

37. Ngo NT, Cao XT, Kneen R, Wills B, Nguyen VM, Nguyen TQ, Chu VT, Nguyen TT, Simpson JA, Solomon T, White NJ, Farrar J, (2001) Acute management of dengue shock syndrome: a randomized double-blind comparison of 4 intravenous fluid regimens in the first hour. Clin Infect Dis 32: 204-213

38. Cifra HL, Velasco, N.J.J. , (2003) A comperative study of the efficacy of 6% Haes-Steril and Ringer's lactate in the managment of dengue shock syndrome. Crit Care Shock 6: 95-100

39. Mutter TC, Ruth CA, Dart AB, (2013) Hydroxyethyl starch (HES) versus other fluid therapies: effects on kidney function. The Cochrane database of systematic reviews: CD007594

40. Simmons CP, McPherson K, Van Vinh Chau N, Hoai Tam DT, Young P, Mackenzie J, Wills B, (2015) Recent advances in dengue pathogenesis and clinical management. Vaccine 33: 7061-7068

41. Panpanich R, Sornchai P, Kanjanaratanakorn K, (2006) Corticosteroids for treating dengue shock syndrome. Cochrane Database Syst Rev: CD003488

42. Tam DT, Ngoc TV, Tien NT, Kieu NT, Thuy TT, Thanh LT, Tam CT, Truong NT, Dung NT, Qui PT, Hien TT, Farrar JJ, Simmons CP, Wolbers M, Wills BA, (2012) Effects of short-course oral corticosteroid therapy in early dengue infection in Vietnamese patients: a randomized, placebo-controlled trial. Clin Infect Dis 55: 1216-1224

43. Nguyen TH, Vu TT, Farrar J, Hoang TL, Dong TH, Ngoc Tran V, Phung KL, Wolbers M, Whitehead SS, Hibberd ML, Wills B, Simmons CP, (2013) Corticosteroids for dengue - why don't they work? PLoS Negl Trop Dis 7: e2592

44. Zhang F, Kramer CV, (2014) Corticosteroids for dengue infection. Cochrane Database Syst Rev: CD003488

45. Whitehorn J, Rodriguez Roche R, Guzman MG, Martinez E, Gomez WV, Nainggolan L, Laksono IS, Mishra A, Lum L, Faiz A, Sall A, Dawurung J, Borges A, Leo YS, Blumberg L, Bausch DG, Kroeger A, Horstick O, Thwaites G, Wertheim H, Larsson M, Hien TT, Peeling R, Wills B, Simmons C, Farrar J, (2012) Prophylactic platelets in dengue: survey responses highlight lack of an evidence base. PLoS Negl Trop Dis 6: e1716

46. Khan Assir MZ, Kamran U, Ahmad HI, Bashir S, Mansoor H, Anees SB, Akram J, (2013) Effectiveness of platelet transfusion in dengue Fever: a randomized controlled trial. Transfusion medicine and hemotherapy : offizielles Organ der Deutschen Gesellschaft fur Transfusionsmedizin und Immunhamatologie 40: 362-368

47. Lye DC, Lee VJ, Sun Y, Leo YS, (2009) Lack of efficacy of prophylactic platelet transfusion for severe thrombocytopenia in adults with acute uncomplicated dengue infection. Clin Infect Dis 48: 1262-1265

48. Thomas L, Kaidomar S, Kerob-Bauchet B, Moravie V, Brouste Y, King JP, Schmitt S, Besnier F, Abel S, Mehdaoui H, Plumelle Y, Najioullah F, Fonteau C, Richard P, Cesaire R, Cabie A, (2009) Prospective observational study of low thresholds for platelet transfusion in adult dengue patients. Transfusion 49: 1400-1411
